# Supplementary figures and images for: Chronic Lymphocytic Leukemia (CLL)-Derived Extracellular Vesicles Educate Endothelial Cells to Become IL-6-Producing, CLL-Supportive Cells
Source: Biomedicines. 2024 Jun 21;12(7):1381. doi: 10.3390/biomedicines12071381 (PMC11273944; doi:10.3390/biomedicines12071381)

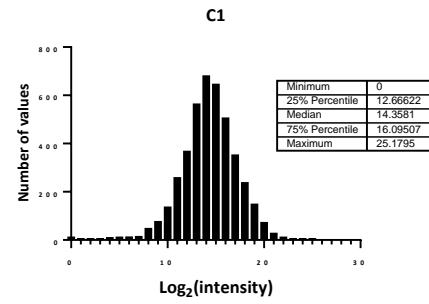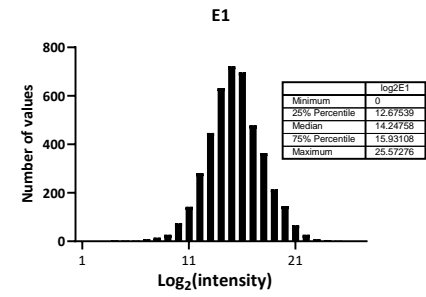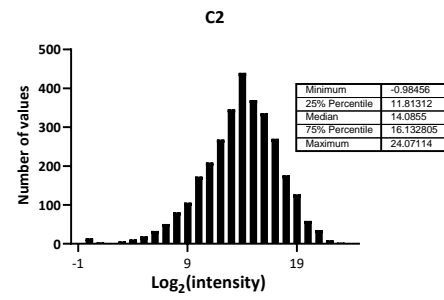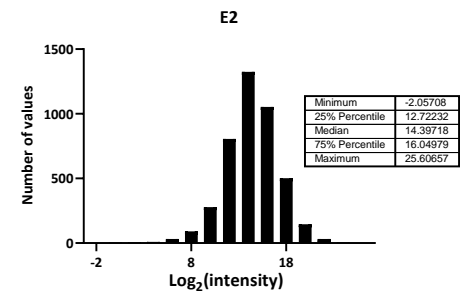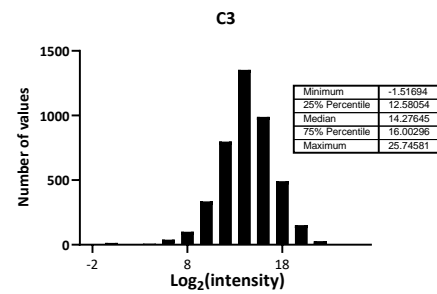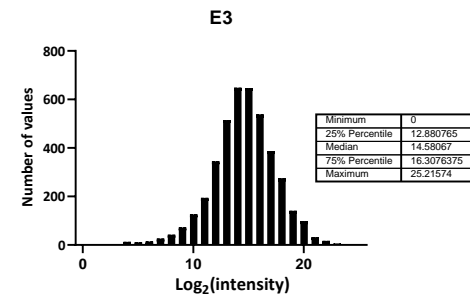

Supplement: Supplementary file 1 [file biomedicines-12-01381-s001.zip › biomedicines-3024911-supplementary.pdf]
